# Effects of Spiritual Well-Being on Caregiver Burden of Parents of Children with Tracheostomy in Türkiye: The Mediating Role of Psychological Resilience

**DOI:** 10.1007/s10943-025-02312-8

**Published:** 2025-04-18

**Authors:** Hamide Nur Çevik Özdemir, Hülya Gürbüz

**Affiliations:** 1 Department of Pediatric Nursing, Faculty of Health Science, Afyonkarahisar Health Sciences University, Afyonkarahisar, Turkey; 2 Department of Pediatric Nursing, Institute of Health Sciences, Ege University, İzmir, Turkey

**Keywords:** Caregiver burden, Children with tracheostomy, Nurse, Resilience, Spirituality

## Abstract

This study aimed to investigate the mediating role of psychological resilience in examining the effects of spiritual well-being on the caregiver burden of parents of children with tracheostomy. This descriptive, cross-sectional, and correlational study was conducted with 109 parents of children with tracheostomy. The three-factor spiritual well-being scale, the caregiver burden scale for family caregivers, and the brief resilience scale were used for data collection. The study adhered to the strengthening the reporting of observational studies in epidemiology guidelines. Descriptive statistics, t tests, variance analysis, and relationship analysis were used in the data analysis. A significant, negative, and moderate relationship exists between the caregiver burden and the brief resilience scale (*p* < 0.05). There is a negative relationship between the anomie sub-dimension of the spiritual well-being scale and caregiver burden, and a positive and moderate relationship with resilience (*p* < 0.05). Psychological resilience affects caregiver burden via the anomie subscale. Programs can be developed to alleviate the caregiving burden and enhance the spiritual well-being of parents with children who have tracheostomies, taking into account the importance of psychological resilience.

## Introduction

Tracheostomy is a surgical procedure to create a safe airway for medical treatment and various indications for congenital or acquired airway obstruction, respiratory failure, and neurological diseases (Sanders et al., 2018; Swain et al., 2021). In recent years, it has been performed more frequently in children due to technological advancements in pediatric intensive care units. Children with tracheostomy are a critical and vulnerable group who rely on medical technology for ongoing treatment and care in their own homes (Sanders et al., 2018; Watters, 2017.)

After completing the hospitalization process of children with tracheostomy, their primary caregivers provided home care (Hartnick et al., 2017). Parents are responsible for complex tasks, such as sputum aspiration, cleaning, and changing the tracheostomy tube during home care (Gong et al., 2019). Parents face physical and emotional distress and increased caregiver burden due to increased home care responsibility (Gong et al., 2019; Özdemir Çevik & Şenol, 2019).

Although parents are adapting to the home care processes of a child with special needs, many of them suffer from health problems, such as caregiver burden, frustration, anxiety, and excessive fatigue (Acorda et al., 2023; Caicedo, 2014; Flynn et al., 2013). Moreover, caregivers of children with chronic illnesses or medical complexity face physical, emotional, social, and economic burdens, such as social isolation, alienation, and financial problems (Koch & Jones, 2018; Özdemir Çevik & Şenol, 2022). Existing studies have reported that parents of children with tracheostomy feel inadequately equipped to manage situations, such as aspiration, infection, tube dislodgement, and seizures, and experience negative emotions, such as anxiety and stress (Gong et al., 2019; Hall et al., 2023; McCormick et al., 2015).

## Spirituality’s Role in Caregiver Burden

Religion and spirituality are acknowledged as powerful coping mechanisms or a source of peace and hope for caregiving parents (Kwon, 2015). Spirituality plays a significant role as a motivating and adaptive force in human life (Hill & Pargament, 2003) and gives meaning to one’s life in times of stress and crisis (Rafati et al., 2020; Rahimi et al., 2013).

The World Health Organization (WHO) recognizes spiritual and mental health as an important part of general health (WHO, 1998). Previous studies have revealed that spiritual well-being is a strong predictor of caregiver burden in chronic diseases (Anum & Dasti, 2016; Rafati et al., 2020; Türkben Polat & Kıyak, 2023; Vigna et al., 2020). However, studies analyzing spirituality in caregivers of children with chronic diseases are quite limited (Abdoljabbari et al., 2018; Anum & Dasti, 2016; Sira et al., 2014). Research indicates that spiritual well-being alleviates the challenges of caregiving for parents, enhancing their psychological resilience and coping strategies (Abdoljabbari et al., 2018; Anum & Dasti, 2016; Tuncay & Sarman, 2024). Considering all these findings, spiritual well-being is thought to affect the caregiver burden and psychological resilience of parents of children with tracheostomy.

Parents should be resilient to cope with the challenges they face (Alsharaydeh et al., 2019). Psychological resilience is expressed as adapting to stressful situations, being able to perform responsibilities despite difficulties, and surviving distress (Smith et al., 2008). A study revealed that parents’ resilience in caring for a child with tracheostomy helped them cope with challenges (Flynn et al., 2023). Psychological resilience is affected by various individual, cultural, and environmental factors (Southwick et al., 2014). Nurses should determine the caregiver burden that may affect the health of caregivers and its associated factors, such as psychological resilience and spiritual well-being (Türkben Polat & Kıyak, 2023). Accordingly, the psychological resilience of parents of a child with a tracheostomy may affect the caregiver burden.

Mediation is a causal model that explains the “why” and “how” of the cause-and-effect relationship between variables. A mediator variable is highly useful for understanding the mechanism through which a cause (independent variable) produces an effect (on the dependent variable) (Fairchild & MacKinnon, 2009). Dey et al. (2021) demonstrated the mediating role of resilience in the relationship between spirituality and subjective well-being among parents of children with special needs. Ginevra et al. (2018) showed that career adaptability is indirectly related to life satisfaction through resilience among parents of children with intellectual disabilities. In light of this information, spiritual well-being may be a protective factor for psychological resilience, which may mediate the effects of spiritual well-being on caregiver burden. Currently, no research has examined how psychological resilience mediates the relationship between spiritual well-being and the caregiver burden of parents of children with tracheostomy.

Therefore, this study aimed to examine the mediating role of psychological resilience in examining the effects of spiritual well-being on the caregiver burden of parents of a child with tracheostomy. This study is also thought to make important contributions to the gap in the literature as it is the first study on the subject and will provide an opportunity to understand the effects of spiritual well-being on parents of children with tracheostomy.

## Study Questions

1. What are the defining characteristics of parents of children with tracheostomies?
2. What are the spiritual well-being, care burden, and psychological resilience levels of parents of children with tracheostomy?
3. What is the relationship between spiritual well-being, caregiver burden, and psychological resilience levels of parents of children with tracheostomy?
4. Is there a relationship between descriptive characteristics and spiritual well-being, caregiver burden, and psychological resilience of parents of children with tracheostomy?
5. Is there a mediating role of psychological resilience in the effects of spiritual well-being on the caregiver burden of parents of children with tracheostomy?

## Methods

### Design

This descriptive, cross-sectional, and correlational study was reported in adherence to the Strengthening the Reporting of Observational Studies in Epidemiology guidelines.

### Study Sample and Participants

The study population consisted of parents of children with tracheostomy residing in a province in western X from January to August 2023. A total of 109 parents of children with tracheostomy met the inclusion criteria (providing home care for a child with tracheostomy for at least 3 months, aged ≥ 18 years, primarily responsible for care, members of the social media group “children with tracheostomy,” open to communication, Internet access, no psychiatric history, no visual impairment, and volunteered to participate in the study). In the post hoc power analysis performed using the G-Power 3.1.9.4 program, the relevant parameters for a sample size of 109 were calculated as effect size 0.236, significance level 0.05, confidence interval 95%, and power 0.95.

### Data Collection Tools

The descriptive information form, the three-factor spiritual well-being scale (SWBS), the caregiver burden scale for family caregivers (CBSFC), and the brief psychological resilience scale (BRS) were used for data collection.

### Introductory Information Form

The researchers prepared the introductory information form in line with the literature (Gong et al., 2019; Hartnick et al., 2017) consisting of a total of 17 questions about the sociodemographic characteristics of the parents (age, gender, educational status, occupation, etc.) and children (age, gender, time of tracheostomy opening, etc.).

### SWBS

Ekşi and Kardaş (2017) developed SWBS to understand the lives of adults and determine their living processes. The scale consists of three factors and 29 items and uses a five-point Likert-type scale. The scale sub-dimensions are transcendence, harmony with nature, rulelessness, and anomie. The sub-dimensions of the scale consisted of items “Transcendence (items: 1, 4, 5, 8, 9, 12, 13, 16, 17, 20, 21, 24, 25, 27, 29),” “Harmony with Nature (items: 2, 6, 10, 14, 18, 22, 28),” and “Anomie (items: 3, 7, 11, 15, 19, 23, 26).” The scale items are responded as 1 “not applicable to me at all” to 5 “completely applicable to me.” The minimum score is 29, and the maximum score is 145 from scale. A high score in each sub-dimension of the scale indicates that the individual possesses the characteristic evaluated by that sub-dimension. The average of the sub-dimensions and the total score is taken when scoring the scale. The Cronbach’s alpha value of the scale was 0.763 (Ekşi & Kardaş, 2017) and that of this study was 0.895.

### CBSFC

CBSFC was developed by Çevik Özdemir and Şenol (2022) to measure the caregiver burden of family caregivers of children with chronic diseases such as cancer. The researchers indicated that the scale can also be used for caregivers of children requiring special care or having chronic diseases (Çevik Özdemir & Şenol, 2022). The scale consists of 36 items and four sub-dimensions (emotional, mental, physical, sociocultural, and economic burdens). Each item on the scale is rated on a five-point Likert scale: 1, never; 2, rarely; 3, sometimes; 4, mostly; and 5, always. The minimum score on the Likert scale is 1 (36*1 = 36 points), and the maximum score is 5 (36*5 = 180 points). The caregiver’s burden is determined by summing up the responses to all the questions in the scale. According to the Likert scale, scores ranging from 1.00 to 2.00 indicate no or minimal burden, 2.00 to 3.00 indicate mild to moderate burden, 3.00 to 4.00 indicate moderate to severe burden, and 4.00 to 5.00 indicate extreme burden. The Cronbach’s alpha value of this scale was determined as 0.93 (Çevik Özdemir & Şenol, 2022) and that of this study was 0.976.

### BRS

Smith et al. (2008) developed a scale to measure the psychological resilience of individuals. The scale is a five-point Likert-type and six-item self-reported instrument. The minimum score is 6 (1 × 6), and the maximum score is 30 (5 × 6). The assessment of the scale was based on the following Likert scale: 1.00–2.99, low resilience; 3.00–4.30, normal resilience; and 4.31–5.00, high resilience. A high score on the scale indicates high psychological resilience. The internal consistency reliability coefficient of the scale varies between 0.80 and 0.91. Doğan (2015) confirmed the Turkish validity and reliability of the scale. The scale has a unidimensional structure, and Cronbach’s alpha value was 0.83 (Doğan, 2015) and that of this study was 0.752.

### Data Collection

Data were collected with a questionnaire form designed using the Google Forms application. Google Forms is an easy-to-use, secure, free online survey, and form-creation program preferred in scientific research (Krishna et al., 2022). A social media platform (WhatsApp group named children with tracheostomy) was used to reach the parents of children with tracheostomy. Data were obtained from parents who participated in the online survey.

Before starting the study, parents registered on the social media platform (WhatsApp group named children with tracheostomy) were informed about the study and invited to participate. Informed consent was obtained from parents who agreed to participate in the study. The questionnaire was sent to the volunteering parents via their e-mails or WhatsApp accounts. The Google survey form was designed to be filled out only once. Thus, multiple responses were prevented. The questionnaires were completed in approximately 10–15 min. A copy of the collected data was stored on an encrypted computer accessible only by the researchers.

### Data Analysis

Data analysis was performed using the IBM Statistical Package for Social Sciences for Windows 25.0 program. Descriptive data were evaluated using frequency, percentage distribution, mean, median, minimum, and maximum values. Normality tests, histograms, Q–Q graphs, and box-plot graphs were used to evaluate the conformity of the data to a normal distribution. The analysis results revealed that all measurements were found to be normal within ± 3 (Hayran, 2011).

Independent sample *t* test, *F* test, Bonferroni multiple comparison test, Pearson correlation analyses, and regression analysis were used for data analyses. In the study, mediation analyses (Hayes, 2018) were also conducted with Process Macro (Model 4). Analyses were conducted to determine the relationships and mediation between variables within the framework of the created model. The results obtained from the model were interpreted using standardized path estimate (*β*) scores and explained variance (*R*^2^) values. In addition, mediation analysis was performed with Process Macro. Statistical analyses were accepted at a 95% CI, and the significance level was set at *p* < 0.05.

### Ethical Principles

Before starting the study, permission from the clinical research ethics committee of a university (protocol no: 2023/1) was obtained. For the WhatsApp group named children with tracheostomy, permission was obtained from the nurse who is the group administrator. Parents were then informed about the study’s purpose, and their consent was obtained. The study adhered to the Declaration of Helsinki. Parents who volunteered were included in the study. No private information of the parents was collected through the Google Forms.

## Results

The characteristics of the parents and their children with tracheostomy who participated in this study are presented in Table 1. The mean age of the parents was 37.68 ± 6.27 years. About 86.2% of the parents were mothers of the child, and 28.4% of them were primary school graduates. Moreover, 68.8% of the parents were housewives, and 15.5% were workers; 53.2% lived in the province, 46.8% had an income lower than their expenses; 85.3% of participants were in nuclear families, and 69.7% had one or two children; 69.7% had been caring for their child with a tracheostomy for > 6 months, and 67% reported difficulty in providing care; and 81.7% received support from other people (spouse, siblings, relatives, etc.) while providing care (Table 1).

**Table 1:** Distribution of parents according to sociodemographic characteristics (*n* = 109)

Characteristics	*n*	%
*Parents*		
Mother	94	86.2
Father	15	13.8
*Parent’s age*		
< = 35	42	38.5
36—45	53	48.6
46 +	14	12.8
*Educational status*		
Primary school	31	28.4
Secondary school	20	18.3
High school	32	29.4
*Undergraduate/graduate*	26	23.9
Occupation		
Housewife	75	68.8
Officer	6	5.5
Worker	17	15.5
Unemployed	11	10.1
*Residence*		
Provincial	58	53.2
Town/village	51	46.8
*Income status*		
< Expenses	51	46.8
= Expenses	42	38.5
> Expenses	16	14.7
*Family type*		
Nuclear family	93	85.3
Extended family	13	11.9
Broken family	3	2.8
*Number of children*		
< = 2	76	69.7
3 +	33	30.3
*Caregiving difficulties*		
Yes	73	67.0
No	36	33.0
*Receiving support while providing care*		
Yes	89	81.7
No	20	18.3
	Mean ± SD	
Parent’s age (year)	37.68 ± 6.27	

*SD* standard deviation

The mean age of the parents’ children with tracheostomy was 6.83 ± 5.78 years, and 64.2% were girls. All children with tracheostomy were connected to medical devices (Table 2).

**Table 2:** Distribution of children with tracheostomy according to sociodemographic characteristics

Characteristics	x̄ ± SD	
Age (year)	6.83 ± 5.78	
	*n*	%
*Gender*		
Girl	39	35.8
Boy	70	64.2
*Age group*		
< = 2 year	27	24.8
3–6 year	43	39.4
7–12 year	14	12.8
13–18 year	25	22.9
*Tracheostomy opening time*		
< 6 month	24	22.0
> 6 month	85	78.0
*Type of medical device*		
Nasogastric tube	59	54.1
Vascular access	17	15.6
Oxygen tube	14	12.8
Mechanical ventilator	19	17.4

*SD* standard deviation

Table 3 presents the Cronbach’s alpha (*α*) coefficients and some descriptive statistics of the CBSFC, BRS scales, and SWBS subscales (transcendence, harmony with nature, anomie) used in this study. The Cronbach’s alpha coefficient values of the scales were > 0.70, indicating the high levels of reliability in these scales. The mean CBSFC and BRS scores were 113.09 ± 36.54 and 18.82 ± 5.02, respectively. The mean scores for spiritual well-being were calculated as follows: transcendence (67.03 ± 9.40); harmony with nature (30.95 ± 3.85); and anomie (21.90 ± 8.12).

**Table 3:** Reliability analysis results and some descriptive statistics for the scales

Scales	_Cronbach’s Alpha (*α*)_	Min	Max	Mean ± SD
CBSFC	0.976	43.00	180.00	113.09 ± 36.54
BRS	0.752	6.00	30.00	18.82 ± 5.02
SWBS	0.895	82.00	144.00	119.88 ± 15.11
Transcendence	0.912	25.00	75.00	67.03 ± 9.40
Harmony with nature	0.933	20.00	35.00	30.95 ± 3.85
Anomie	0.901	7.00	35.00	21.90 ± 8.12

*CBSFC* caregiving burden scale for family caregivers, *BRS* brief resilience scale, *SWBS* spiritual well-being scale, *Min–Max* minimum–maximum, *SD* standard deviation

CBSFC, Kaiser–Meyer–Olkin measure of sampling adequacy (KMO) = 0.909; Bartlett’s test of sphericity: Chi-square (*χ*^2^) = 4130.606, *p* < 0.001, SWBS, KMO = 0.841, *χ*^2^ = 2408.677, *p* < 0.001, BRS; KMO = 0.717, *χ*^2^ = 215.219, *p* < 0.001

Table 4 shows the Pearson correlation analysis results regarding the relationship between the variables used in the study. There is a statistically significant, inversely, and moderate relationship between the caregiver burden scale and the psychological resilience scale (*r* = −0.501; *p* = 0.000). There is a statistically significant positive, significant, and moderate relationship between the spiritual well-being scale and the psychological resilience scale (*r* = 0.503; *p* = 0.000). There is a inversely and moderate relationship between the anomie dimension and caregiver burden (*r* = −0.592; *p* = 0.000); there is a positive and moderate relationship between the anomie dimension and psychological resilience (*r* = 0.654; *p* = 0.000).

**Table 4:** Correlation coefficients for the relationship between CBSFC, BRS, and subscale of SWBSs

Scales		CBSFC	BRS	SWBS	Transcendence	Harmony with nature	Anomie
CBSFC	r	1					
	p						
BRS	r	−0.501**	1				
	p	0.000					
SWBS	r	−0.300**	0.503**	1			
	p	0.002	0.000				
Transcendence	r	−0.005	0.170	0.816**	1		
	p	0.962	0.077	0.000			
Harmony with nature	r	0.083	0.180	0.638**	0.559**	1	
	p	0.393	0.061	0.000	0.000		
Anomie	r	−0.592**	0.654**	0.614**	0.096	0.066	1
	p	0.000	0.000	0.000	0.323	0.497	

**The correlation is significant at *p* < 0.05. *CBSFC* caregiving burden scale for family caregivers, *SWBS* spiritual well-being scale, *BRS* brief resilience scale

Table 5 depicts the t test and analysis of variance results for the comparison of parents’ SWBS subscales, CBSFC, and BRS levels according to sociodemographic characteristics. Based on the age of the parents, the difference in caregiver burden, psychological resilience, and anomie scores was significant (*p* < 0.05). Anomie, caregiver burden, and psychological resilience scores of parents aged 46 and over are higher than other age groups. A statistically significant difference was found in the transcendence subscale of spiritual well-being between mothers and fathers (*p* = 0.056, borderline significance). Accordingly, the transcendence scores of mothers were found to be higher than those of fathers. However, no significant difference was found according to the type of parent in terms of caregiver burden, psychological resilience, and other subscales of spiritual well-being (*p* > 0.05) (Table 5).

**Table 5:** Comparison of parents’ CBSFC, SWBS subscales, and BRS levels according to sociodemographic characteristics

Variable	Group	CBSFC	BRS	SWBS		
				Transcendence	Harmony with nature	Anomie
		x̄ ± SD	x̄ ± SD	x̄ ± SD	x̄ ± SD	x̄ ± SD
Parent’s age	< = 35 ^(1)^	113.81 ± 31.62	18.93 ± 4.18	67.1 ± 8.61	30.74 ± 4.12	22.26 ± 7.28
	36–45 ^(2)^	105.57 ± 36.33	19.77 ± 5.05	66.43 ± 10.55	31.06 ± 3.78	23.11 ± 7.9
	46 + ^(3)^	139.43 ± 40.96	14.86 ± 5.59	69.07 ± 6.97	31.21 ± 3.53	16.21 ± 9.49
	*F* test	5.135	5.815	0.433	0.115	4.313
	*P*	0.007*	0.004*	0.649	0.892	0.016*
	Bonferroni	**2 < 3**	**3 < 1,2**			**3 < 1,2**
Parent type	Mother	112.78 ± 36.68	18.87 ± 5.13	67.71 ± 8.92	31.06 ± 3.78	22.36 ± 8.22
	Father	115.07 ± 36.8	18.47 ± 4.34	62.73 ± 11.39	30.27 ± 4.38	19 ± 7.01
	*t* test	−0.224	0.290	1.930	0.742	1.497
	*P*	0.823	0.773	0.056	0.460	0.137
Number of children	< = 2	109.08 ± 34.74	19.58 ± 4.66	66.18 ± 10.41	31.11 ± 3.89	22.54 ± 8.23
	3 +	122.33 ± 39.37	17.06 ± 5.43	68.97 ± 6.19	30.61 ± 3.8	20.42 ± 7.79
	*t* test	−1.757	**2.465**	−1.731	0.619	1.253
	*P*	0.082	0.015*	0.087	0.537	0.213
Caregiving difficulties	Yes	126.1 ± 32.92	17.74 ± 4.7	66.36 ± 10.32	30.95 ± 3.7	20.52 ± 7.75
	No	86.72 ± 28.67	21 ± 4.99	68.39 ± 7.1	30.97 ± 4.21	24.69 ± 8.25
	*t* test	**6.120**	**−3.339**	−1.202	−0.034	**−2.589**
	*P*	0.000*	0.001*	0.232	0.073	0.011*
Receiving support while providing care	Yes	112.15 ± 36.89	18.53 ± 4.75	66.78 ± 9.76	30.96 ± 3.77	22.13 ± 7.83
	No	117.3 ± 35.54	20.1 ± 6.03	68.15 ± 7.66	30.95 ± 4.33	20.85 ± 9.46
	*t* test	−0.568	−1.270	−0.589	0.005	0.628
	*P*	0.571	0.207	0.557	0.996	0.525

*P < 0.05, *t* independent sample *t* test, *F* one-way analysis of variance (*F* test). Bold values are the significance value

*CBSFC* caregiving burden scale for family caregivers, *SWBS* spiritual well-being scale, *BRS* brief resilience scale

The caregiver burden and spiritual well-being subscales scores did not show a statistically significant difference based on the number of children of the participants (*p* > 0.05). However, the difference in psychological resilience scores was statistically significant according to the number of children, and those with one or two children (*X* = 19.58) had higher scores than those with three or more children (Table 5).

In Table 5, the caregiver burden, psychological resilience, and spiritual well-being subscales scores of the parents showed a statistically significant difference based on caregiving difficulty (*p* < 0.05). Accordingly, the caregiver burden scores of individuals with caregiving difficulties were found to be significantly high (*p* < 0.05). In addition, it was determined that the anomie subscale scores of psychological resilience and spiritual well-being were lower in individuals experiencing caregiving difficulties.

According to Model 1 in Table 6, psychological resilience has a significant inversely correlated on caregiver burden directly (*β* = −0.1545, *p* < 0.05) and through spiritual well-being (*β* = −0.7251, *p* < 0.05). Since the confidence interval of the mediation effect is (−0.9654 to −0.2443), it was determined that spiritual well-being plays a partial mediation role. These findings show that the effect of psychological resilience on caregiver burden is both direct and mediated by spiritual well-being. In Model 4, the role of anomie in the effect of psychological resilience on caregiver burden was examined. The effect of anomie on caregiver burden (β = −2.6619, *p* < 0.0001) and the direct effect of psychological resilience (*β* = −2.0762, *p* < 0.0001) were found to be significant. These findings show that psychological resilience directly affects caregiver burden, while anomie does not play a mediating role (Table 6).

**Table 6:** BRS mediator role in the relationship between SWBS subscales and CBSFC

Model	Mediating variable	Effect		βeta	Std. Error	t	*p*	Significance
**1**	BRS	SWBS → CBSFC	Effect	−0.251	0.2230	−3.2515	0.0015*	Accept
			Direct effect	−0.1545	0.2348	−0.6581	0.000*	Accept
			Indirect effect	Confidence interval (−0.9654–0.2443)				Yes
**2**	BRS	Transcendence → CBSFC	Effect	−0.0182	0.3759	−0.0483	0.9615	No
			Direct effect	0.3224	0.3302	0.9764	0.3311	No
			Indirect effect	Confidence interval (−0.9845–0.0118)				No
**3**	BRS	Harmony with nature → CBSFC	Effect	0.7836	0.9132	0.8581	0.3928	No
			Direct effect	1.6938	0.7931	2.1355	**0.0350**	Accept
			Indirect effect	Confidence interval (−2.1037–0.0751)				No
**4**	BRS	Anomie → CBSFC	Effect	−2.6619	0.3506	−7.5925	0.000*	Accept
			Direct effect	−2.0762	0.4576	−4.5375	0.000*	Accept
			Indirect effect	Confidence interval (−1.3462–0.1481)				No

*p < 0.05, *CBSFC* caregiving burden scale for family caregivers, *SWBS* spiritual well-being scale, *BRS* brief resilience scale

## Discussion

To the best of our knowledge, this is the first study to demonstrate the mediating effects of psychological resilience on the effects of spiritual well-being on the care burden among parents of children with tracheostomy. Although the study results reveal the need for practices to strengthen the spiritual well-being among caregivers of children with tracheostomy, they also contribute to the gap in the field.

In this study, it was concluded that the caregiving burden, spiritual well-being, and psychological resilience levels of parents with tracheostomized children were at a moderate level. Similarly, Çevik Özdemir and Şenol (2022) reported that the families of children with cancer had a moderate level of caregiving burden, while Karakaya and Kamışlı (2024) stated that the mothers of children diagnosed with chronic illnesses experienced a caregiving burden ranging from moderate to severe levels. Furthermore, Koliouli et al. (2022) emphasized that parents of children with epilepsy turned to spiritual resources for empowerment, while Keniş-Coşkun et al. (2020) revealed a moderate relationship between resilience and caregiving burden among parents of pediatric rehabilitation patients. These findings suggest that interventions aimed at reducing the caregiving burden and supporting the spiritual well-being of parents of tracheostomized children could enhance their psychological resilience and may also be effective for similar patient groups.

This study revealed that caregiver burden, psychological resilience, and anomie scores differed as parents’ age groups of parents. Parents in middle adulthood (≥ 46 years) had a higher caregiver burden and lower psychological resilience and spiritual well-being than parents in other age groups. A previous study reported that the caregiver burden is higher among families of medically complex children (Baddour et al., 2021). Another study demonstrated that as the age of mothers caring for children with cerebral palsy increased, their care burden increased and spiritual orientation decreased (Tuncay & Sarman, 2024). A study conducted on caregivers of patients suffering from spinal cord injuries revealed that more resilient caregivers perceived lower care burdens (Castellano-Tejedor & Lusilla-Palacios, 2017). Our research results were consistent with those in previous studies. With the increased age of caregivers, child care may become a more challenging problem. Physiological changes related to aging may increase the care burden of individuals. Therefore, attention should be paid to the physical health of caregivers in middle adulthood, and proactive assistance should be provided.

In our study, while caregiver burden and psychological resilience were not affected by the gender of the parents, the spiritual well-being of mothers was significantly higher than that of the fathers. Tuncay and Sarman (2024) found that mothers with a high spiritual orientation had less caregiver burden. Studies indicating that spiritual well-being is related to gender have reported that women have higher spiritual well-being than men (Hammermeister et al., 2005; Yilmaz, 2019). These results emphasize the impact of gender on spiritual well-being. Social norms and culture effectively shape gender roles, which can have different effects on spiritual well-being. Considering that mothers take a greater role in caring for their children and are more likely to provide emotional support, they can also have higher spiritual well-being. Further studies are needed to reveal gender differences in spiritual well-being and underlying causes.

In this study, parents with one or two children had higher psychological resilience than those with three or more children. A study on parents caring for a child with cancer revealed that the number of children had an impact on parents’ psychological resilience and spiritual well-being (Koyu et al., 2024). This can be associated with the increased number of individuals they have to care for at home, changes in parents’ family roles, and increased responsibilities toward their children. As the caregiver’s responsibility increases, the caregiving process becomes tiring and long (Çevik Özdemir & Şenol, 2022). Low resilience of parents with chronically ill children will lead to the development of psychological morbidity in parents (Rothschild et al., 2020). Intervention studies to increase psychological resilience may provide additional proactive support for high-risk parents. Therefore, future studies focusing on specific interventions for parents with low psychological resilience are needed.

This study is consistent with the existing literature and emphasizes that both psychological resilience and spiritual well-being directly and inversely correlated with parents’ caregiver burden levels (Newberry et al., 2013; Rafati et al., 2020; Türkben Polat & Kıyak, 2023). In our study, with the increased caregiver burden of parents, their spiritual well-being and psychological resilience decreased. Spirituality and religious traditions provide family caregivers with a sense of purpose and meaning in challenging situations while relieving the burden of caregiving responsibilities (Pearce, 2005; Yilmaz, 2019). Spirituality is a cost-effective and efficient strategy that can alleviate the caregiver burden (Rafati et al., 2020). A study on caregivers of non-ambulatory children reported that caregiver burden was related to resilience (Keniş Coşkun et al., 2020). Another study revealed that more resilient caregivers of patients with spinal cord injury experienced lower caregiver burdens (Castellano-Tejedor & Lusilla-Palacios, 2017). Nurses should enhance the coping strategies and promote the psychological resilience of parents caring for a chronically ill child at home with targeted interventions (Li et al., 2022).

Our study revealed that caregivers’ spiritual well-being was positively correlated with psychological resilience. Likewise, the increased psychological resilience of parents of chronically ill children also increased their spiritual well-being (Koyu et al., 2024; Luo et al., 2022). One study documented that having high resilience positively affected caregivers’ mental health (Anderson et al., 2020). Another study indicated that a spiritual program for caregivers of children with autism increased resilience among parents (Pandya, 2018). Spirituality helps individuals cope with challenges more effectively by increasing their ability to cope with stress and thereby increasing psychological resilience. The parents in our study may have turned to more spiritual resources (prayer, worship, etc.) to cope with stress. However, further research is warranted to better understand what influences the psychological resilience of parents caring for their homebound children.

The current study’s findings indicate that psychological resilience mediates the impact of parents’ spiritual well-being on caregiving burden. This finding aligns with studies by Dey et al. (2021), Pandya (2018), and Amin et al. (2024), suggesting that spirituality may enhance parents’ resilience to their children’s illnesses. Spirituality plays an important role in building resilience, coping with challenges, and improving well-being (King et al., 2006). The results of our study reveal that spiritual well-being and psychological resilience are effective factors in the caregiver burden of parents of a child with a tracheostomy.

## Study Limitations

This study had some limitations. First, because this study was cross-sectionally designed, true causal inferences could not be interpreted. Longitudinal and interventional studies are needed to confirm the findings. Second, because this study was carried out exclusively with parents enrolled on a specific social media site, the findings cannot be applied to other demographics. Third, data were collected through self-reported questionnaires, which may contain bias due to over- or underestimation.

Finally, the SWBS used in this study is not only a measurement tool focusing on spiritual well-being, but also provides a structure that evaluates general well-being (existential) and spiritual well-being together. Caution should be exercised when using SWBS due to the potential for combined subscale contamination, and therefore, results may be tautological and misleading (Bambling, 2024; Koenig & Carey, 2024). Subscales need to be reported separately. However, there are several reasons why the SWBS was chosen for this study. First, the SWBS provides a widely used and validated measure in a variety of cultural contexts for many years. In addition, attention was paid to the similarity of the culture in which the psychological scales used in this study were developed and the culture in which they were applied. Being aware of the limitations of the SWBS, the analysis of the subscales separately is intended to preserve the validity of the results obtained. This study included the appropriate separate analysis of the subscales to ensure the validity of the results.

## Conclusion

This study found that caregiver burden was associated with parents’ age and caregiving difficulty. Furthermore, variables such as gender and number of children were associated with spiritual well-being and psychological resilience. Psychological resilience affects caregiving burden through the anomie sub-dimension of spiritual well-being. Based on the findings of our research, it seems important to pay attention to the spirituality of parents in the process of caring for their children. This can help increase their psychological resilience. Therefore, it seems that the healthcare team consisting of nurses, doctors, and psychologists who provide care to children with tracheostomies and their families should pay attention to the spiritual needs of families and plan spiritual care programs.

Nurses and home care teams should regularly assess the spiritual well-being, psychological resilience, and caregiver burden of caregivers of children with tracheostomy. In future studies, it is suggested that specialized family support systems be developed to alleviate caregiving burden and intervention programs related to spiritual well-being and resilience be created.

## Relevance to Clinical Practice

Our study revealed that parental factors may be influential on caregiver burden. The results give clues for clinical practice and provide evidence for the need to develop supportive systems (psychological support services, stress management, etc.) for parents of children with tracheostomy. These findings can help understand the challenges faced by parents of children with tracheotomies and develop effective interventions. Based on the results, intervention studies involving mental well-being and resilience are needed to effectively reduce the burden on caregivers.

Pediatric nurses play an important role in planning interventions that will reduce parents’ caregiving burden and support their spirituality and psychological resilience. The findings of this study will guide intervention programs planned in pediatric units and home care services to support spirituality and psychological resilience in parents of children with tracheostomies. These programs should be planned with particular consideration for the dimensions of psychological resilience and spiritual well-being.
